# Developing a High-Umami, Low-Salt Soy Sauce through Accelerated Moromi Fermentation with *Corynebacterium* and *Lactiplantibacillus* Strains

**DOI:** 10.3390/foods13091386

**Published:** 2024-04-30

**Authors:** Li-Hao Wang, Wen-Hui Qu, Ya-Nan Xu, Song-Gang Xia, Qian-Qian Xue, Xiao-Ming Jiang, Hong-Ying Liu, Chang-Hu Xue, Yun-Qi Wen

**Affiliations:** 1 College of Food Science and Engineering, Ocean University of China, 1299 Sansha Road, Qingdao 266400, China; daozhangwang23@gmail.com (L.-H.W.); quwh9959@163.com (W.-H.Q.); xuyanan152@163.com (Y.-N.X.); songgang_xia@163.com (S.-G.X.); xue851668452@163.com (Q.-Q.X.); jxm@ouc.edu.cn (X.-M.J.); oucxuech@163.com (C.-H.X.); 2 Qingdao Institute of Marine Bioresources for Nutrition & Health Innovation, Qingdao 266109, China; 3 Ocean College, Hebei Agriculture University, Qinhuangdao 066000, China; liu066000@sina.com

**Keywords:** umami sauce, rapid fermentation, low salt, moromi fermentation

## Abstract

The traditional fermentation process of soy sauce employs a hyperhaline model and has a long fermentation period. A hyperhaline model can improve fermentation speed, but easily leads to the contamination of miscellaneous bacteria and fermentation failure. In this study, after the conventional koji and moromi fermentation, the fermentation broth was pasteurized and diluted, and then inoculated with three selected microorganisms including *Corynebacterium glutamicum*, *Corynebacterium ammoniagenes*, and *Lactiplantibacillus plantarum* for secondary fermentation. During this ten-day fermentation, the pH, free amino acids, organic acids, nucleotide acids, fatty acids, and volatile compounds were analyzed. The fermentation group inoculated with *C. glutamicum* accumulated the high content of amino acid nitrogen of 0.92 g/100 mL and glutamic acid of 509.4 mg/100 mL. The *C. ammoniagenes* group and *L. plantarum* group were rich in nucleotide and organic acid, respectively. The fermentation group inoculated with three microorganisms exhibited the best sensory attributes, showing the potential to develop a suitable fermentation method. The brewing speed of the proposed process in this study was faster than that of the traditional method, and the umami substances could be significantly accumulated in this low-salt fermented model (7% *w*/*v* NaCl). This study provides a reference for the low-salt and rapid fermentation of seasoning.

## 1. Introduction

Soy sauce is consumed regularly in East Asia, which is made from soybean and wheat, and it contains abundant umami components, such as glutamine, aspartate, IMP, GMP, succinic acid, etc. Additionally, soy sauce has a rich, distinctive odor due to the volatile organic compounds, such as aldehydes, alcohols, and esters. Traditional soy sauce production requires considerable amounts of salt. However, a high-salt diet is related to hypertension [1] and some kidney metabolism disease [2]. China, South Korea, and Thailand have enacted policies to reduce the salt content in umami-flavored condiments to reduce the health risk of excessive salt intake [3]. Therefore, a new production process for preparing low-salt soy sauce was explored in this study.

Using a high salt level (around 18–20% *w*/*v* NaCl) during fermentation could inhibit the proliferation of harmful bacteria [4,5]. However, the excessive salt content also restricts the growth and metabolization of fermentative bacteria, leading to the extended period for traditional soy sauce fermentation, meaning the long fermentation cycle and high production cost [6]. In addition, the style of product obtained from high-salt fermentation is difficult to diversify. Therefore, reducing the salt in fermented umami seasonings such as soy sauce and fish sauce is proposed. However, this fermentation mode makes it difficult to effectively inhibit the growth of harmful bacteria, which could produce unpleasant odors [7,8]. Meanwhile, low salinization is reported to be detrimental to accumulating pleasant volatiles during fermentation by Chun et al. [9]. Nanofiltration and electrodialysis technology are applied in the desalination of soy sauce, but the compound compositions of treated sauce change greatly compared with initial sauce [10,11].

*C. glutamicum* is capable of producing significant quantities of glutamic acid [12]. As *C. glutamicum* has a relatively clear genetic background, it can be transformed into a related strain for biodesign synthesis. It is also considered as safely edible, so it is commonly used in the food industry, for example, the synthesis of branched chain amino acids [13]. *C. ammoniagenes* can produce nucleotides under suitable conditions [14]. *L. plantarum* has the potential to be a probiotic [15], with a variety of potential health benefits, such as metabolites that have the potential to lower cholesterol in humans [16]. The food safety of *L. plantarum* has been established [15], and it can utilize soybean and its protein [17]. *L. plantarum* can produce lactic acid during the fermentation process, which can perform as a preservative and reduce the amount of food additives [18].

During the production of umami condiments, such as soy sauce, big sauce, etc., soybean is first fermented into koji under the enzyme system of *Aspergillus oryzae* [19], followed by the moromi process, during which lactic acid bacteria and yeast play an important role and produce flavor compounds [20,21]. The moromi fermentation requires a substantial amount of salt and has a longer fermentation time.

High-salt-concentration fermentation can inhibit the proliferation of *C. glutamicum*, *C. amoniagne*, and *L. plantarum*. When the salt level is suitable, the metabolisms of these three microorganisms recover, and these three microorganisms may conduct quick fermentation. In addition, when multiple bacterial strains are combined in food fermentation, they may produce certain probiotic effects.

In this study, the fermentation process of koji was first carried out for 48 h, and then the moromi fermentation process was carried out for fifteen days with a salt concentration of 14% *w*/*v* NaCl. Subsequently, the fermentation broth was sterilized, diluted, and inoculated with *C. glutamicum*, *C. ammoniagenes*, and *L. plantarum* for secondary fermentation. By measuring the changes in pH, amino acid nitrogen, sensory, free amino acids, taste activity value (TAV), organic acids, nucleotides, fatty acids, and volatile compounds of the fermentation broth, this study explored the impact of secondary fermentation on the quality of soy sauce. This study aimed to develop a low-salt and rapid fermentation method of soy sauce production accompanied by producing rich umami substances, which could provide a reference for the rapid industrial production of soy sauce.

## 2. Materials and Methods

### 2.1. Chemicals

Soybean meal and wheat were purchased from China COFCO Group (Beijing, China). *Aspergillus oryzae* was provided by Qingdao Dengta Brewage Co., Ltd. (Qingdao, China). Standard products were purchased from Shanghai Aladdin Biochemical Technology Co., Ltd. (Shanghai, China). *Corynebacterium glutamicum* (CICC^®^10046), *Corynebacterium ammoniagenes* (CICC^®^20168), and *Lactiplantibacillus plantarum* (CICC^®^10481) were purchased from the China Center of Industrial Culture Collection (CICC, Beijing, China).

### 2.2. Fermentation of Soy Sauce

**Figure 1:**
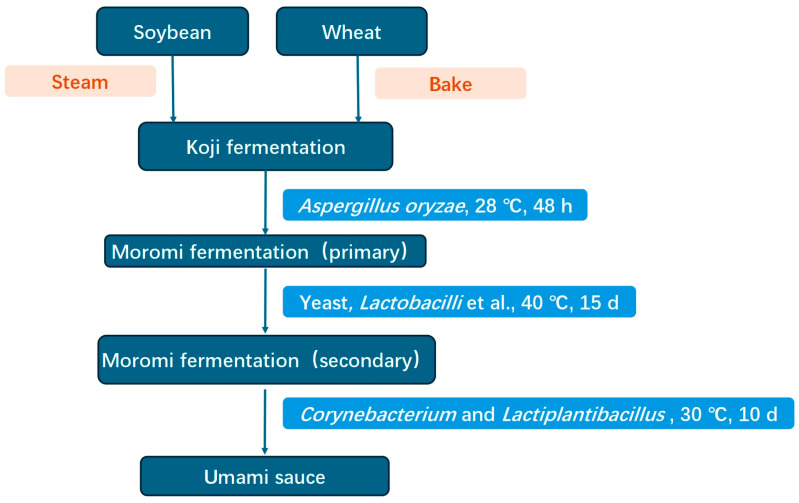
The flow chart of the whole fermentation process of soy sauce [19,22].

The flow chart of the fermentation process of soy sauce is shown in Figure 1.

#### 2.2.1. Fermentation Process of Koji and Moromi (Primary Fermentation)

Soybeans were soaked in water for 6 h and cooked using a pressure cooker with saturated steam for 40–45 min. The wheat was baked in an oven at 150 °C for 60 s. The prepared soybeans were mixed with wheat at a mass ratio of 1:1. *Aspergillus oryzae* spores were inoculated for fermentation with a mass fraction of 0.05% *w*/*v*. Fermentation was performed at 90% relative humidity and 28 °C until the appearance of yellow–green spores. This process needed about 48 h. Then, brine with the concentration of 14% *w*/*v* NaCl was added for moromi fermentation at 40 °C for 15 days. This process was dominated by natural bacteria, mainly including yeast and lactic acid bacteria. During the moromi fermentation, the pH was approximately 6.4 at the initial stage and 6.0 at the end.

#### 2.2.2. Secondary Fermentation of Moromi by Three Selected Strains

After 15 days of moromi fermentation, the fermentation broth was sterilized using the pasteurization method (60 °C, 30 min) and then inoculated with *Corynebacterium glutamicum*, *Corynebacterium ammoniagenes*, and *Lactiplantibacillus plantarum* for secondary fermentation to accumulate umami substances.

The processes of inoculation and fermentation refer to Li et al. [22] and are described as follows. *Corynebacterium glutamicum*, *Corynebacterium ammoniagenes*, and *Lactiplantibacillus plantarum* were inoculated onto MRS solid culture medium and incubated at 30 °C. After one day, the single colonies were introduced into MRS liquid culture medium at 30 °C for cultivation with stir at 225 rpm. Bacterial liquid sample was taken every 1 h for OD600 testing and the growth curve was drawn to obtain bacterial cells at the exponential growth phase. After centrifugation (8000× *g* for 2 min), the bacterial cells were collected and mixed with isotonic saline to suspend the bacterial liquid. Then, the bacterial cells were inoculated into fermentation medium, i.e., the mixture of moromi and water, in which the moromi and water were mixed at a volume ratio of 1:1. Finally, the salt concentration of fermentation broth declined from 14% to 7% and the concentration of the bacteria in fermentation medium was approximately 10^6^ CFU/mL. Group 1 was inoculated with *C. glutamicum*. Group 2 was inoculated with *C. ammoniagenes*. Group 3 was inoculated with *L. plantarum*. Group 4 was inoculated with the mixed bacterial solution containing three types of bacteria. The inoculation process was carried out on an ultra-clean bench and the fermentation broth was sealed before secondary fermentation at 30 °C for 10 days. Sampling was conducted at 0, 1, 3, 5, 7, and 10 days during the fermentation phase.

### 2.3. Sensory Assessment

A group of ten people (seven female/three male; aged between 21 and 39 years) was recruited from the Ocean University of China (Qingdao, China) for sensory analysis. To accurately identify the taste and odor of fermentation broth, the group received training for 10 days before the experiments. The odor vocabularies and attributes describing sample were confirmed through referring to Diez-Simon et al. [23] and the discussion of panelists. Finally, umami, salty, sweet, bitter, and sour were selected for taste analysis, and caramel, fruity, malty, nutty, mushroom, and roasty were picked up for odor. The sensory evaluation was carried out in a sensory panel room kept at 23 ± 2 °C. An amount of 5 g sample was placed into a 100 mL glass vessel that was covered and presented to each panelist. The intensity of each taste or odor attribute was evaluated using a 0–10 point scale with 0.5 steps; 1 min rest was necessary for sensory recovery.

### 2.4. Amino Acid Nitrogen Content Analysis

Five grams of fermentation product was dissolved in ultrapure water. The solution was diluted to a final volume of 50 mL and 1 mL was pipetted into 10 mL colorimetric tube. After that, 4 mL of colorimetric solution (37% methanol and 7.8% acetylacetone solution) and sodium acetate–acetic acid buffer (pH 4.8) were added and ultrapure water were replenished to 10 mL. The mixed solution was kept in boiling water for 15 min. After cooling, the absorbance at 400 nm was measured. External standard method was used for quantification.

### 2.5. Free Amino Acid Analysis

Five grams of fermented sauce sample were mixed with 20 mL of pre-cooled 5% ethanol, and incubated for 30 min at 4 °C, then centrifuged at 12,000× *g* for 5 min at 4 °C. The supernatant was transferred to a new centrifuge tube and thoroughly dried by nitrogen gas. Then, 5 mL of 0.02 N hydrochloric acid was used for dissolution. This sample was filtered through 0.22 μm syringe filters before subjecting to an automatic amino acid analyzer (L-8800, Ltd. 6-6, Marunouchi 1-chome, Chiyoda-ku, Tokyo, Japan).

The taste activity value (TAV) was determined as reported by Kato et al. [24]. The TAV reflects the contribution of compounds to the taste profile. The higher the value, the bigger the contribution of taste. According to Duan et al. [25], the TAV was calculated as follows:

TAV = *C*_1_/*C*_2_

The concentration of flavor of amino acid is denoted as *C*_1_, the value of *C_2_* indicates the threshold concentration and the value of *C*_2_ refers to the book titled “Flavour Thresholds: Compilations of Flavour Threshold Values in Water and Other Media” [26].

In order to more intuitively present the impact of concentration changes of amino acid on taste during the fermentation, this study used TAV variation for evaluation. The calculation formula is as follows:

TAV variation = TAV_t_ − TAV_0_

In the formula, TAV_t_ represents the TAV of the amino acid at the fermentation time t, and TAV_0_ represents the TAV of the same amino acid at the beginning of fermentation.

### 2.6. Determination of Nucleotide

Five gram samples were homogenized using 15 mL of pre-cooled 5% perchlorate, and incubated for 30 min at 4 °C, then centrifuged at 10,000× *g* for 5 min at 4 °C. The supernatant was mixed with 15 mL of pre-cooled 5% perchloric acid again, shaken, and incubated for 30 min at 4 °C, and the supernatant was obtained through centrifugation. The pH of the supernatant was adjusted to 6.7. The nucleotide analysis was conducted by HPLC system (Agilent Technologies Co., Ltd., Palo Alto, CA, USA) consisting of a 1680 UV detector and column (150 mm × 4.6 mm, 5 μm). The mobile phase was prepared by water (pH 4.8) containing 0.02 mol/L citric acid, 0.02 mol/L glacial acetic acid, and 0.04 mol/L triethylamine. Samples were detected at 260 nm with the flow rate at 1.0 mL/min, and the column temperature was kept at 40 °C. Qualitative analysis was conducted with co-eluted chromatographic standards and external standards curves were used for quantitative analysis.

### 2.7. Determination of Organic Acids

Organic acids were determined based on a modified version described by Chen et al. [27]. A five gram sample and 10 mL of 0.05 mol/L disodium hydrogen phosphate solution were mixed in a centrifuge tube, shaken, and incubated for 30 min at 4 °C. The mixture was centrifuged at 12,000× *g* for 10 min at 4 °C. The supernatant was mixed with 10 mL of 0.05 mol/L disodium hydrogen phosphate solution again to the precipitate. The above operation was repeated and the supernatant was collected. Subsequently, the sample was passed through 0.22 μm filter for HPLC analysis. An Agilent 1680II (Agilent Technologies Co., Ltd., Palo Alto, CA, USA) coupled with a Waters 120-5-C18 AQ column (150 mm × 4.6 mm, 5 μm) was adopt. Deionized water containing 0.05 mol/L disodium hydrogen phosphate (pH 3.0) was used as mobile phase with a flow rate at 1.00 mL/min. The detection wavelength was 210 nm, and the column temperature was set at 25 °C. External standards curves were used for quantitative analysis.

### 2.8. Fatty Acids Composition Analysis

The fermented sample was extracted by chloroform/methanol (2/1, *v*/*v*) and separated through centrifugation to obtain the total lipid. Then, fatty acid methyl esters were prepared referring to Wen et al. [28]. A 7890a GC-MS instrument (Agilent Technologies, Santa Clara, CA, USA) equipped with an HP-5MS capillary column (30 m × 0.25 mm × 0.20 μm) (Agilent Technologies, Santa Clara, CA, USA) was used. The initial temperature was set at 80 °C, then it was increased to 200 °C at 20 °C per min, and then to 280 °C at 5 °C/min. Finally, the temperature was increased to 300 °C at 10 °C/min and held at 300 °C for 5 min. The temperature of the ion source was set at 260 °C and the mass spectrometer was operated in EI mode at 70 eV with a filament current of 25 μA.

### 2.9. Determination of Volatile Organic Compounds

Analysis of the volatile organic compounds of samples from different fermentation stages was performed using an SHS–GC–IMS system referring to Wen et al. [29], The samples (0.5 g) were transferred into a 20 mL headspace glass sampling vial and incubated at 45 °C for 30 min. After incubation, 0.5 mL of headspace gas samples was automatically injected into the injector under splitless injection mode with a syringe at 80 °C. Nitrogen (99.99% purity) was used as the carrier gas at the following programmed flow: initially 2 mL/min for 2 min, increased to 15 mL/min in 8 min, increased to 100 mL/min in 10 min, and increased to 150 mL/min in the last 10 min. The total detection time was 30 min. Purified nitrogen (99.99% purity) was set as the drift gas for IMS at a flow rate of 150 mL/min, and the drift tube temperature was set to a constant temperature of 45 °C.

### 2.10. Statistical Analysis

All results were presented as the mean ± standard deviation (SD) from triplicate separate and independent measurements or testing. Statistical analysis was performed using SPSS 21 (IBM, New York, NY, USA). One-way ANOVA was used for calculating statistical significance, and Duncan’s test for the 5% significance level.

## 3. Results and Discussion

### 3.1. Change in pH and Amino Acid Nitrogen Content during Fermentation

**Figure 2:**
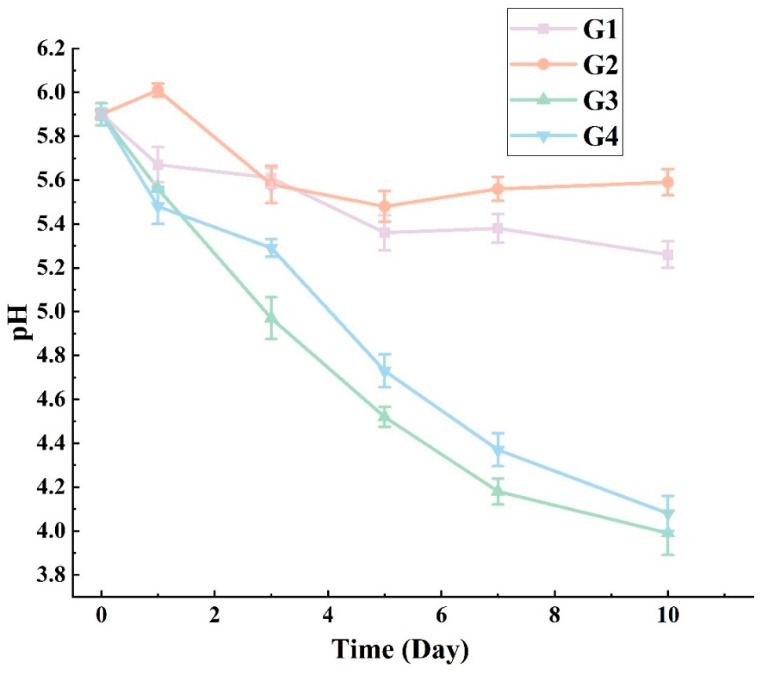
The pH trends of G1, G2, G3, G4 during secondary fermentation of moromi. G1: the group inoculated with *Corynebacterium glutamicum*; G2: the group inoculated with *Corynebacterium ammoniagenes*; G3: the group inoculated with *Lactobacillus plantarum*; G4: the group inoculated with above three strain.

The pH value of fermentation broth was related to the transformation of reducing sugar to acid. The pH levels of samples during the 10 days of secondary fermentation are shown in Figure 2. When only *C. glutamicum* (G1) or *C. ammoniagenes* (G2) participated in fermentation, the pH decreased slightly, and finally reached 5.25 and 5.59, respectively. When *L. plantarum* was used, the pH decreased significantly, and finally decreased to 3.98 (G3) and 4.10 (G4). These results show that *L. plantarum* is an important participant in reducing sugar metabolism during fermentation, which is consistent with Cui et al. [30]. In addition, the pH trends of G3 and G4 were similar, indicating *L. plantarum* could still effectively convert sugars into acids although it was mixed with other strains.

Hu et al. [8] fermented soy sauce at the salt concentration of 9% for 40 days. Dominated by *Bacillus* and *Kurthia*, the pH eventually dropped to 4.25. Yang et al. [31] fermented broad bean paste at low salt (11%), and the pH dropped to 4.4 after 50 days of fermentation. Seiji et al. [32] suggested that when moromi was fermented in low-salt conditions, the pH decreased more rapidly than that in high-salt conditions, and the main bacteria involved in the fermentation were *Lactiplantibacillus* and *Bacillus*. In this study, the moromi was pasteurized before the secondary fermentation and only three selected strains participated, meaning the environment was more suitable for their growth and *L. plantarum* could quickly metabolize reducing sugar.

The pH of the fermentation broth decreased slightly when it only contained *C. glutamicum* in this study, perhaps because *C. glutamicum* uses the phosphoenolpyruvate-dependent sugar phosphotransferase system (PTS) to take up sugar [33]. PTS is less efficient when multiple carbon sources are used simultaneously, such as when glucose is supplied with acetate, then the glucose absorption is reduced [34], and when glucose and gluconate become carbon sources at the same time, the utilization efficiency of glucose further decreases [35], and the pathway has potential conflict with the ability to produce amino acids, meaning the low utilization efficiency may be related to the various non-single-phase glycogen provided by moromi [36].

**Figure 3:**
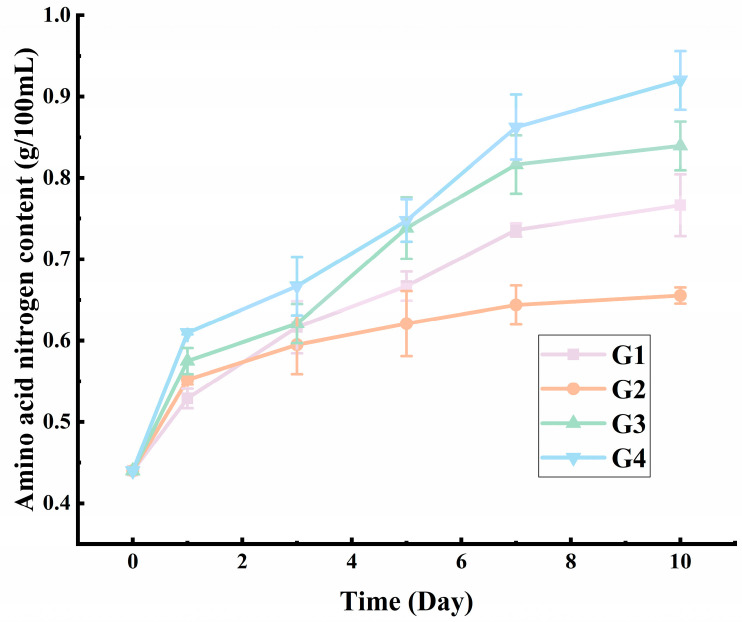
The amino acid nitrogen content (g/100 mL) of G1, G2, G3, G4 during secondary fermentation of moromi at 37 °C for ten days. G1: inoculated with *Corynebacterium glutamicum*; G2: inoculated with *Corynebacterium ammoniagenes*; G3: inoculated with *Lactobacillus plantarum*; G4: inoculated with above three strains.

Amino acid nitrogen is an important quality indicator of umami seasoning. The dynamic content of amino acid nitrogen in each group during the fermentation is shown in Figure 3. The amino acid nitrogen contents of the four groups all increased during fermentation. Among them, the amino acid nitrogen of G2 (*C. glutamicum* group) showed the slowest increase and finally reached 0.60 g/100 mL. The amino acid nitrogen of G1 finally reached 0.71 g/100 mL. G3 (*L. plantarum* group) increased fast and finally reached 0.83 g/100 mL. As for G4, the amino acid nitrogen finally reached 0.92 g/100 mL, reaching the first level of China’s national soy sauce standard.

In the low-salt solid-state fermentation process of soy sauce by Zhang et al. [37], the amino acid nitrogen reached 0.7 g/100 mL. Additionally, during the low-salt fermentation of doubanjiang (broad bean paste) with the salt concentration of 11%, the amino acid nitrogen reached 1.0 g/100 mL after 50 days [31]. Hu et al. [8] fermented soy sauce and the final amino acid nitrogen was about 0.25 g/100 mL, suggesting that the fermentation of moromi using environmental microorganisms may be difficult in low-salt conditions.

As the salinity selected in this study was lower than that of traditional condiments, during the ten days of fermentation, the initial increase rate of amino acid nitrogen content was high and then it gradually decreased. Interestingly, when using *L. plantarum* alone for fermentation, the amino acid nitrogen showed the fastest increased, which was significantly different from the other two bacteria.

### 3.2. Sensory Change during Fermentation

**Figure 4:**
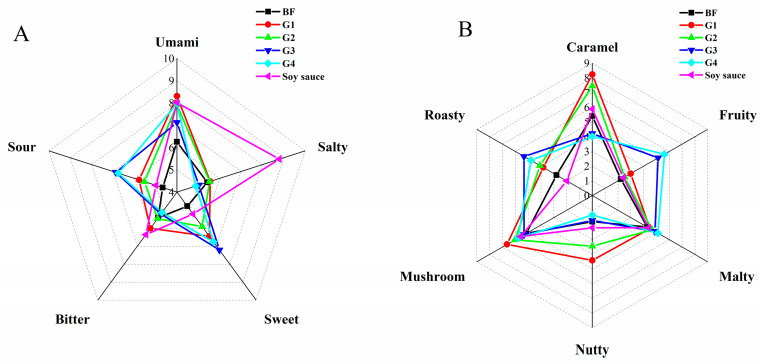
Sensory taste (**A**) and odor (**B**) profiles of G1–G4, BF, and commercial soy sauce. The odor and taste intensities represented by the score are as follows: 0: not perceptible, 2: slightly perceptible, 4: perceptible, 6: considerably perceptible, 8: strongly perceptible, and 10: very strongly perceptible. BF: the moromi before inoculation and fermentation; G1: inoculated with *Corynebacterium glutamicum*; G2: inoculated with *Corynebacterium ammoniagenes*; G3: inoculated with *Lactobacillus plantarum*; G4: inoculated with above three strains.

The taste and odor of the four fermentation groups and commercial soy sauce were evaluated and comparation. As shown in Figure 4A, the umami taste of the four fermentation groups all increased when compared with moromi before the secondary fermentation (BF) (6.2), in which the scores of G1, G2, G3, and G4 were 8.3, 7.9, 7.1, and 8.0, respectively. The salty scores of G1–G4 were lower than that of the commercial soy sauce (8.8). The bitter scores of the four fermentation groups were slightly lower than that of the commercial soy sauce. The sour scores of the groups added with *L. plantarum* (G3, G4) increased significantly from 4.7 (BF) to 6.9 and 6.7, respectively. The sweet taste of G3 and G4 was stronger than other groups. Due to the high acid production of *L. plantarum*, the sour taste of the groups with *L. plantarum* (G3, G4) was more obvious, and the taste profile was quite different from that of the groups without *L. plantarum*. According to Feng et al. [38], compared with high-salt soy sauce, low-salt soy sauce could have a stronger sourness and lower sweetness. *C. glutamicum* and *C. ammoniagenes* could increase umami taste during soy sauce fermentation, which is related to the accumulation of amino acids and succinic acid [39,40]. *L. plantarum* could increase the sour and sweet, resulting in a different flavor of fermentation product, which is related to the accumulation of organic acids [30].

In terms of odor (Figure 4B), the caramel and nutty scores in the *C. glutamicum* group (G2) were higher than those in the BF group, which were 8.2 and 4.4, respectively, while the fruity score in G3 and G4 was significantly high, with the scores of 5.13 and 5.62, respectively. The malty scores of all groups were similar. The odor profiles of G1 and G2 were close, and the groups with the participation of *L. plantarum* (G3, G4) were close.

The fruity and roasty aroma are important flavors for soy sauce [41]. *L. plantarum* fermentation enhanced both the flavor of fruity and roasty. Hu et al. [8] reported that if the traditional moromi fermentation was carried out in the low-salt pattern, the harmful bacteria would increase, leading the odor and taste to deteriorate.

### 3.3. The Free Amino Acid Compositions during Fermentation

Asp and Glu are important umami amino acids in umami condiments, and Thr, Ser, Gly, and Ala are sweet amino acids. Val, Met, Ile, Leu, Tyr, Phe, His, and Arg are bitter amino acids. The content changes of free amino acids during the fermentation are shown in Table 1. For G1 samples, the contents of Asp increased from 83.1 mg/100 mL to 139.6 mg/100 mL, and the content of Glu increased from 215.9 mg/100 mL to 509.4 mg/100 mL. The content of Thr and Ala increased from 208.3 mg/100 mL and 293.1 mg/100 mL to 366.6 mg/100 mL and 438.6 mg/100 mL, respectively. The Asp and Glu contents in the G2 increased from 82.8 mg/100 mL and 219.2 mg/100 mL to 107.2 mg/100 mL and 459.4 mg/100 mL, respectively, and both final contents were lower than those in G1. However, after fermentation, Lys, Val, Ile, Leu, and Phe in G2 all had the highest contents among the four groups, reaching, respectively, 340.8 mg/100 mL, 620.8 mg/100 mL, 744.1 mg/100 mL, 1213.4 mg/100 mL, and 774.2 mg/100 mL. The Asp concentration in G3 was the highest among the single-bacteria fermentation groups, reaching 171.0 mg/100 mL. Glu finally reached 419.2 mg/100 mL, which was lower than that in G1 and G2. The contents of bitter amino acids Ile, Leu, Tyr, and Phe in G3 were much lower than those in G1 and G2. The Tyr content in G3 was the highest, which led to the difference in fermentation characteristics between G3 and the other groups. The contents of Asp and Glu in the mixed fermentation group (G4) finally reached 169.8 mg/100 mL and 474.5 mg/100 mL, respectively. During the secondary fermentation, free amino acids were rapidly accumulated, especially the umami amino acids and sweet amino acids. The accumulation of Glu, Ala, and Thr in G1, the accumulation of Lys, Val, and Pro in G2, and the accumulation of Asp and Tyr in G3 were more abundant than that in other groups.

L-Glutamic acid is synthesized by a one-step enzymatic reaction catalyzed by glutamate dehydrogenase from the tricarboxylic acid (TCA) cycle intermediate 2-oxloglutarate [42]. L-Lysine, L-isoleucine, and L-aspartic acid were synthesized from the same TCA cycle intermediate oxaloacetate [43]. The metabolic levels of Glu, Asp, Ala, Pro, Lys, and His could be influenced by environment [44,45,46]. It could be seen that some amino acids showed no significant content change during the fermentation, such as the Tyr in G1 and G2, possibly because these free amino acids did not participate in the fermentation and metabolism process [47].

**Figure 5:**
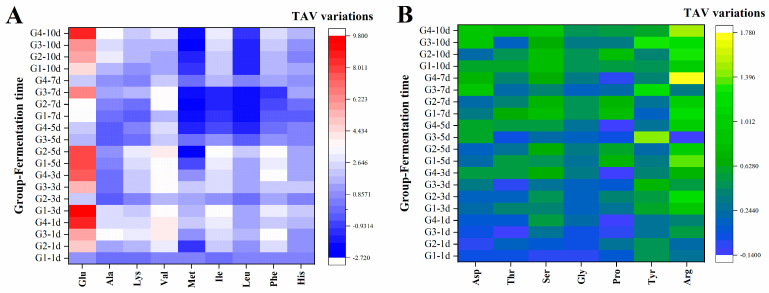
(**A**,**B**) The variation of taste activity value (TAV) of 16 amino acids from G1–G4 at different fermentation stages. G1: inoculated with *Corynebacterium glutamicum*; G2: inoculated with *Corynebacterium ammoniagenes*; G3: inoculated with *Lactobacillus plantarum*; G4: inoculated with above three strains. The fermentations time were marked afterwards, for example, “−1 d” means fermentation for one day.

Based on the calculation of TAV and the variations of TAV, Figure 5 presents the changes in the contribution of 16 amino acids to the taste of soy sauce during fermentation. It should be mentioned that the negative value of TAV variation indicates that the contribution of amino acid to the taste of soy sauce is weakened after fermentation. After 10 days fermentation, the amino acids in G1 that had great impact on taste were Glu, Val, Ile, Phe, and Ala, with the TAV variations values of 9.8, 3.6, 3.4, 2.9, and 2.4, respectively. For G2, the amino acids that had a great impact on taste were Glu, Val, Phe, Ile, and Lys, with the values of 8.1, 4.2, 3.8, 3.5, and 2.7, respectively. In G3, affected by the fermentation of *L. plantarum*, the amino acids that had a greater impact on the taste were Glu, Val, Leu, Met, and Ile. The corresponding values were 6.8, 3.6, −2.3, −2.2, and −1.8. In the mixed bacteria fermentation group (G4), Glu, Ala, Ile, Val, and Phe were the amino acids that had a greater impact on taste, with values of 8.6, 3.4, 3.0, 2.5, and 2.2, respectively.

It was obvious that the amino acid with greatest impact on taste was glutamic acid, which was reflected in G1, G2, G3, and G4, with variations value of 9.8, 8.1, 6.8, and 8.6, respectively. In addition to Glu, Ala was sweet amino acid and the influence of Ala on taste exceeded the sum of Thr, Ser, and Gly in the four groups. The umami amino acid Asp, the sweet bitter amino acids Pro and Tyr, and the bitter amino acid Arg showed limited impact on the taste during fermentation.

### 3.4. Changes in Flavor Nucleotides during Fermentation

**Figure 6:**
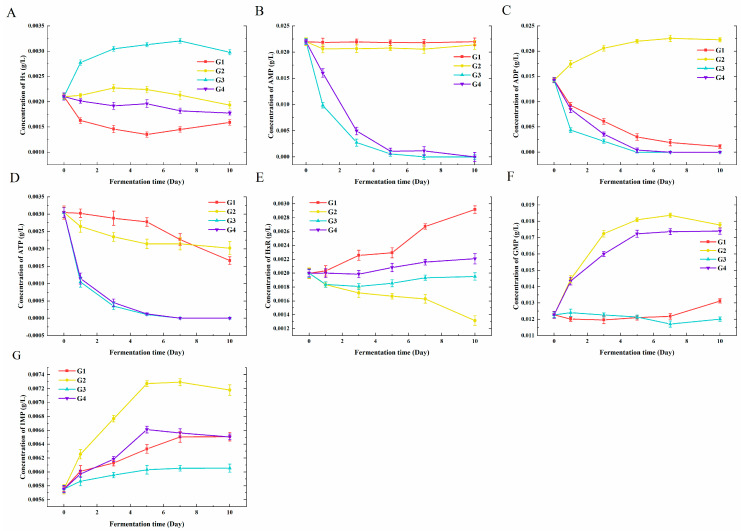
The nucleotide contents of G1–G4 at different stages of 10 day fermentations. (**A**) Hx, (**B**) AMP, (**C**) ADP, (**D**) ATP, (**E**) HxR, (**F**) GMP, and (**G**) IMP. G1: inoculated with *Corynebacterium glutamicum*; G2: inoculated with *Corynebacterium ammoniagenes*; G3: inoculated with *Lactobacillus plantarum*; G4: inoculated with above three strains.

As shown in Figure 6A, G3 occupied the highest content of Hx (2.8 mg/L) after fermentation among four groups, and other groups showed slight decreases in Hx content. The AMP content of G1 and G2 had no significantly change during the fermentation, but it dropped from 22.0 mg/L to 0 mg/L in both G3 and G4. The ADP in G2 increased from 14.3 mg/L to 22.3 mg/L. However, its contents in G1, G3, and G4 declined to 1.1 mg/L, 0 mg/L, and 0 mg/L, respectively. The ATP content showed a downward trend during fermentation, among which G1 and G2 dropped from 3.0 mg/L to 1.7 mg/L and 2.0 mg/L, respectively, and G3 and G4 dropped to 0 mg/L. Though the fermentation of *L. plantarum* (G3), the contents of AMP, ADP, and ATP were all reduced to undetectable levels. The HxR contents showed discrepant trends in the four groups with the initial content of 2.0 mg/L. After fermentation, HxR contents in samples varied from high to low: G1, G4, G3, and G2 with 2.9 mg/L, 2.2 mg/L, 1.9 mg/L, and 1.3 mg/L, respectively.

Flavor nucleotides impart unique flavor to soy sauce, and I+G (IMP plus GMP) is an important index to evaluate soy sauce [48]. *C. glutamicum* and *C. ammoniagenes* have the potential to ferment and produce nucleotide substances [49]. It was obvious that the GMP content in G2 and G4 had been improved from 12.2 mg/L to 17.1 mg/L and 16.4 mg/L, respectively. But the content changes in G1 and G3 were not obvious, which indicates that *C. ammoniagenes* might play a role in the production of GMP. The content of IMP increased significantly in G2 and finally reached 7.2 mg/L, as well as reaching 6.5 mg/L, 6.1 mg/L, and 5.5 mg/L in G1, G3, and G4, respectively. During the fermentation of G2, the contents of IMP and GMP gradually increased, but the increase in IMP content was lower than expected, which might be related to the restriction of glycolytic pathway [50]. Compared to industrial strains of *C. ammoniagenes* that produce nucleotides, the ability of the strains in this experiment to produce nucleotides may be less than expected due to differences in promoter or epitope modifications [14].

In general, the fermentation of *L. plantarum* was not conducive to the accumulation of nucleotide substances, and although *C. ammoniagenes* had a certain accumulation of nucleotide substances, the fermentation did not produce a large amount of nucleotide substances as expected, among which the increase in IMP and GMP contents were relatively limited. *C. glutamicum* and *C. ammoniagenes* did not efficiently accumulate flavor nucleotides, which may be their inefficient utilization of reducing sugar metabolism in the environment [36]. The proper sugar content could increase fermentation rate and yield I + G, etc.

### 3.5. Changes in Organic Acids during Fermentation

**Figure 7:**
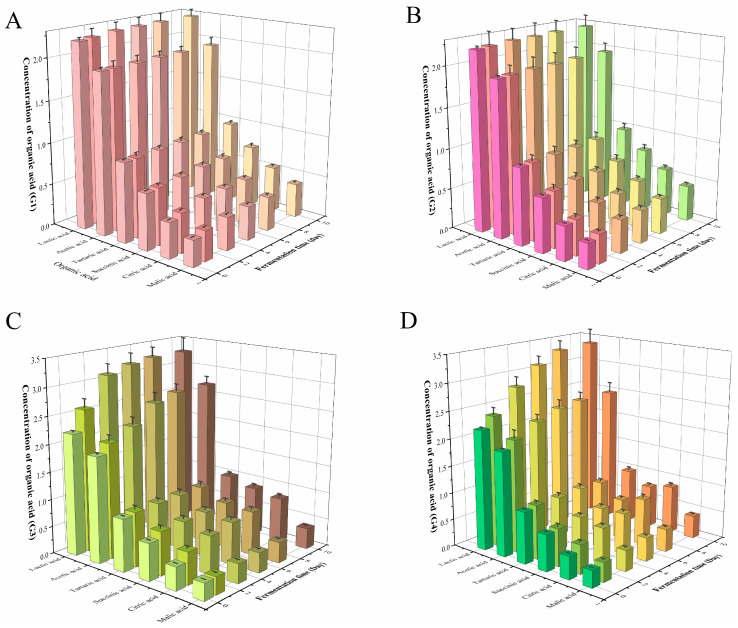
The contents of six organic acids in G1–G4 at different stages of 10 days fermentation, including lactic acid, acetic acid, tartaric acid, succinic acid, citric acid, and malic acid. (**A**) G1, (**B**) G2, (**C**) G3, and (**D**) G4. G1: inoculated with *Corynebacterium glutamicum*; G2: inoculated with *Corynebacterium ammoniagenes*; G3: inoculated with *Lactobacillus plantarum*; G4: inoculated with above three strains.

As shown in Figure 7, the contents of all measured organic acids in G1 and G2 improved, among which malic acid and citric acid showed the most significant increase. In G1 and G2, citric acid increased from 0.42 g/L to 0.54 g/L and 0.58 g/L, respectively, and malic acid increased from 0.31 g/L to 0.41 g/L and 0.44 g/L, respectively. G3 accumulated organic acids significantly during the fermentation process, in which lactic acid increased from 2.20 g/L to 3.28 g/L, acetic acid increased from 1.92 g/L to 2.68 g/L, citric acid increased from 0.42 g/L to 0.81 g/L, and succinic acid increased from 0.67 g/L to 0.88 g/L. The accumulation of organic acids was higher than that in G1 and G2. The contents of organic acids in G4 increased obviously. The contents of citric acid, malic acid, lactic acid, acetic acid, and succinic acid increased, respectively, to 216%, 141%, 156%, 130%, and 127% of the original contents after 10 days fermentation.

Zhang et al. [51] found that the citric acid, lactic acid, and malic acid in moromi y increased by 24.6%, 15.3%, and 0.05%, respectively, in the fermentation by *L. plantarum*, which were similar to this study. Under the combined action of yeast and *Lactiplantibacillus plantarum*, the lactic acid and acetic acid in tomato sauce increased to 10.2 g/L and 3.6 g/L, respectively, during the fermentation cycle, indicating the production potential of organic acids during the fermentation of *L. plantarum* [52].

Compared with high-salt fermentation, low salinity is considered to be more conducive to acid production, and as the concentrations of organic acids increase, the pH decreases. The contents of malic acid, citric acid, and succinic acid may be related to the regulation of glycolysis [53]. During the fermentation process, *L. plantarum* could produce a large amount of lactic acid through the Embden–Meyerhof–Parnas (EMP) pathway and simultaneously produces citric acid [54], which may explain the large accumulation of lactic acid in G3 and G4. The reducing sugars in the environment were converted into organic acids, which was reflected in the decrease in pH and the improvement in sour taste in sensory evaluation.

### 3.6. Changes in Volatile Compounds during Fermentation

**Figure 8:**
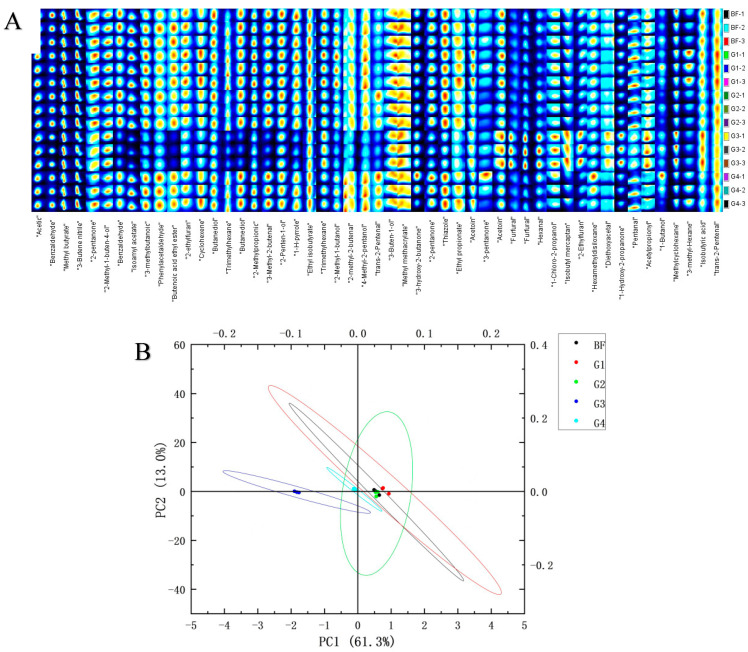
HS–GC–IMS analysis of volatiles from BF and G1–G4 after ten days of fermentation. Analyses were performed in triplicate. (**A**) HS–GC–IMS fingerprints of volatile substances. (**B**) PCA score plot of the data for volatile substances. BF: the moromi before inoculation and fermentation; G1: inoculated with *Corynebacterium glutamicum*; G2: inoculated with *Corynebacterium ammoniagenes*; G3: inoculated with *Lactobacillus plantarum*; G4: inoculated with above three strains.

The volatiles of fermentation samples from different periods are shown in Figure 8A. A total of 51 compounds were detected, including 4 acids, 12 alcohols, 10 aldehydes, 1 alkene, 8 ketones, 2 furan, 5 alkane, and 3 other compounds. Alcohols, aldehydes and ketones are mainly formed through the oxidative metabolism of proteins and fats during fermentation [55]. Benzaldehyde, methyl butyrate, and acetoin were detected in all groups. Benzaldehyde has a special almond smell and is an important component of cherry smell [56]. Methyl butyrate produces an apple aroma, and acetoin has a pleasant buttery aroma.

According to the principal component analysis (PCA) (Figure 8B), the volatile substances of G1 and G2 were different from that of G3. The contents of phenylacetaldehyde, 2-ethylfuran, butanediol, 2-pentanone, and pentanal in G1 and G2 were higher than those in G3. Phenylacetaldehyde has the aroma of hyacinth, and after dilution it has the sweet aroma of fruit [57]. 2-Pentanone presents in the metabolism of cheese fermentation [58]. The discrepancy between BF and G1–G4 indicates that the selected three strains influence the fermented volatile compounds, especially reflected in the volatile of G3. Some volatiles accumulated in high amounts in G3, such as furfural, hexanal, diethoxy acetal, and acetyl propionyl. Furfural inspires almond flavor [59], and diethoxy acetal is the main flavoring ingredient in some distilled alcoholic beverages, especially malt whiskey and sherry [60].

### 3.7. Change in Fatty Acid Compositions during Fermentation

**Figure 9:**
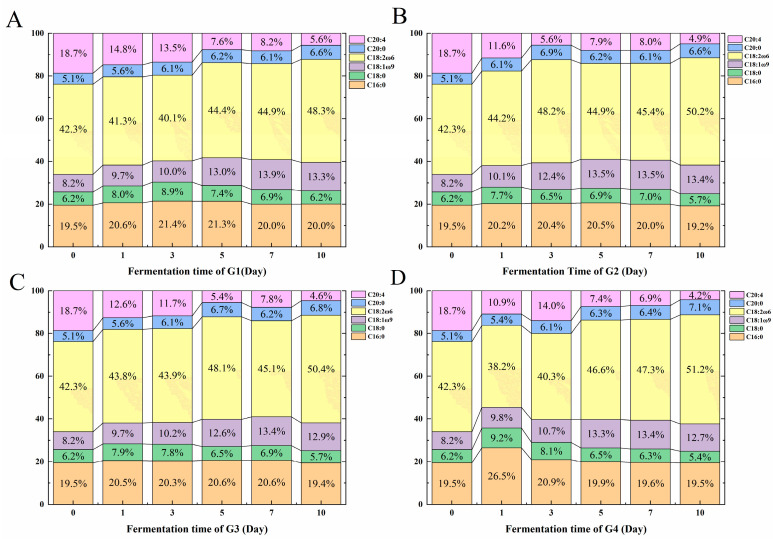
The fatty acid compositions of G1–G4 at different stages of 10 days fermentation, including C16:0, C18:0, C18:1ω9, C18:2ω6, C20:0, and C20:4. (**A**) G1, (**B**) G2, (**C**) G3, (**D**) G4. G1: inoculated with *Corynebacterium glutamicum*; G2: inoculated with *Corynebacterium ammoniagenes*; G3: inoculated with *Lactobacillus plantarum*; G4: inoculated with above three strains.

The fatty acid contents of G1–G4 during fermentation are shown in Figure 9. The C16:0 contents of G1–G4 showed no significant change during fermentation. Similarly, the C18:0 only had slight decrease in G2–G4 after fermentation from 6.2% to 5.7%, 5.7%, and 5.4%, respectively. In contrast, the C18:1ω9 and C18:2ω6 contents of the four groups increased at the end of fermentation. The C18:2ω6 was the most abundant fatty acid in the moromi, and its proportions in G1–G4 increased from 42.3% to 48.3%, 50.2%, 50.4%, and 51.2%, respectively. As to C20:4, it had a significant decrease in the four groups. Among them, G4 showed the largest content decline from 18.4% to 4.2%. According to Zou et al. [61], during the 90-day fermentation of soy sauce, the proportion of C18:2ω6 as the dominant fatty acid increases from 48.07% to 50.55%. The proportion of C16:0 decreases from 15.73% to 14.77%, and the proportion of C18:0 decreases from 6.45% to 5.54%, and the proportion of C18:1ω9 increases from 11.79% to 12.42%.

During the fermentation, the proportion of oleic acid and linoleic acid increased, which might be related to the synthesis of microorganisms. Some key genes, such as *FAS-I* and *FasR* type fatty acid synthases, show the possibility of the synthesis pathway of fatty acid by *C. glutamicum* [62,63] and the mutants of *fasR20*, *fasA63*, and *fasA2623* also cause *C. glutamicum* to accumulate oleic acid and palmitic acid [64]. The fatty acid compositions during the fermentation by *L. plantarum* may be affected by the concentration of alcohols in the environment, thereby reducing the proportion of unsaturated fatty acids [65].

## 4. Conclusions

This study established a low-salt and rapid fermentation process of soy sauce. Prepared moromi underwent pasteurization and was diluted with water to reduce its salt content to 7% *w*/*v*, followed by the inoculation with selected microorganisms and cultivated in a sealed aseptic environment for secondary fermentation within 10 days. After the fermentation, all groups had increases in the umami and sweetness, as well as the caramel flavor, which was different from that of commercially available soy sauce. The umami substance accumulated quickly, especially glutamic acid, which was the most significant factor improving the umami flavor. The group inoculated with *C. glutamicum* occupied the highest amino nitrogen content of 0.92 g/100 mL and glutamic acid content of 509.4 mg/100 mL. Furthermore, the group inoculated with three microorganisms displayed a unique flavor profile that might be useful for fermenting new seasoning.

This fermentation method provided a reference for the rapid fermentation of fish sauce or other condiments, and the produced low-salt soy sauce was beneficial in reducing human salt intake and disease risks, and could enrich the types of umami condiments. The new fermentation method can shorten the fermentation cycle and reduce the usage of salt, which can improve the production efficiency of soy sauce, facilitate the control of fermentation process, and may decrease the production cost when used in the food industry. However, due to the low salt, the fermentation process of this method was susceptible to the influence of stray bacteria leading to fermentation failure. Therefore, avoiding contamination should be strictly performed during fermentation. In future study, more kinds of microorganisms can be considered for participation in fermentation to produce unique or more abundant flavors of soy sauce. Meanwhile, other kinds of materials, for example, gluten powder, can be mixed with soybean for the co-fermentation.

## Figures and Tables

**Table 1:** The free amino acid contents of G1–G4 with different fermentation time (mg/100 mL).

G-FD	Asp	Glu	Thr	Ser	Gly	Ala	Lys	Pro	Val	Met	Ile	Leu	Tyr	Phe	His	Arg
G1-0	83.1 ± 0.8 a	215.9 ± 3.7 a	208.2 ± 5.1 a	145.8 ± 2.8 a	97.8 ± 0.8 a	293.1 ± 3.7 a	205.3 ± 5.1 a	328.8 ± 3.4 a	453.4 ± 11.2 a	104 ± 0.7 b	456.2 ± 10.6 a	847.1 ± 7.6 a	105.4 ± 1.2 a	442.3 ± 7.3 a	59.6 ± 1.5 a	124.3 ± 3.6 a
G1-1	85 ± 3.8 a	238.7 ± 15.6 a	206.3 ± 8.5 a	138.8 ± 6.3 a	88.5 ± 6.2 b	298 ± 20.2 a	207.3 ± 9.1 a	351.2 ± 18.1 a	467.2 ± 15.1 a	116.9 ± 4.0 c	473 ± 32.2 a	910.2 ± 41.6 b	147.1 ± 5.9 d	487 ± 26.1 b	71.2 ± 2.6 b	137.5 ± 4.4 b
G1-3	103 ± 7.6 b	362.1 ± 30.7 b	306.2 ± 16.1 b	202.8 ± 16.6 b	114.3 ± 7.2 c	377 ± 19.1 b	284.9 ± 14.3 b	418.7 ± 38.9 b	575.5 ± 26.3 b	63.6 ± 3.7 a	648.7 ± 43.5 b	1019.6 ± 59.4 c	157.7 ± 9.4 e	654.3 ± 60.4 c	80.2 ± 5.3 c	166.4 ± 7.2 c
G1-5	102.6 ± 4.5 b	394.7 ± 19.4 c	342.6 ± 25.0 c	220.4 ± 21.9 c	129.9 ± 11.4 d	483.8 ± 42.0 c	342.7 ± 15.4 c	541.2 ± 39.0 c	621.4 ± 38.2 bc	124 ± 10.1 c	669.5 ± 41.2 b	1189.9 ± 62.2 d	134.8 ± 6.0 c	777.4 ± 46.1 e	79 ± 4.2 c	194 ± 8.8 d
G1-7	119.8 ± 4.3 c	477 ± 15.4 d	379.5 ± 35.3 d	260.6 ± 8.9 d	155 ± 13.7 e	454.7 ± 27.7 c	322.9 ± 32.4 d	558.4 ± 24.4 d	618 ± 29.4 c	171.2 ± 5.9 e	708 ± 32.9 c	1055.4 ± 113.2 c	121 ± 11.3 b	647 ± 32.5 c	95.8 ± 8.8 d	182.7 ± 6.6 cd
G1-10	139.6 ± 9.0 d	509.4 ± 63.5 e	366.6 ± 15.2 d	262.6 ± 14.3 d	158.6 ± 6.4 e	438.6 ± 39.7 d	306.3 ± 9.0 e	492.3 ± 27.3 d	598.9 ± 21.9 c	158.1 ± 14.9 d	759.8 ± 79.4 d	1109.4 ± 42.4 e	146.9 ± 6.6 d	707.7 ± 35.0 d	97.8 ± 3.7 d	176 ± 5.9 c
G2-0	82.8 ± 0.6 b	219 ± 2.0 a	205.6 ± 4.5 a	145.8 ± 3.0 a	98.9 ± 2.0 a	290.1 ± 7.0 a	204 ± 3.1 a	328.2 ± 6.7 a	458.8 ± 9.2 a	103.8 ± 2.5 c	456.4 ± 4.1 a	841.8 ± 15.6 a	106.1 ± 1.6 a	443.6 ± 5.2 a	59.7 ± 0.7 a	124.3 ± 3.9 a
G2-1	78.7 ± 3.1 a	283.8 ± 11.6 b	239.1 ± 9.4 b	162 ± 11.7 b	102.8 ± 7.7 a	271.3 ± 20.1 a	232.7 ± 12.2 b	404.7 ± 15.1 b	522.6 ± 31.0 b	139.2 ± 9.5 e	544.7 ± 29.1 b	865.2 ± 63.4 a	150.4 ± 11.0 d	556.4 ± 39.8 b	66 ± 3.2 b	133.9 ± 4.5 c
G2-3	95.2 ± 7.6 c	372.8 ± 25.4 c	252.9 ± 19.9 b	211.6 ± 18.4 c	120.2 ± 10.8 b	281.5 ± 24.6 a	301.6 ± 28.5 c	450.7 ± 36.6 c	610 ± 53.5 d	165.9 ± 8.7 f	664 ± 31.4 c	1048.3 ± 60.5 b	154.4 ± 12.4 d	642.3 ± 46.4 c	97.7 ± 8.5 e	182.5 ± 3.9 cd
G2-5	99.1 ± 9.2 d	438.1 ± 27.9 d	273.4 ± 16.3 c	242.8 ± 18.3 d	146.1 ± 12.2 c	286.9 ± 26.4 a	310 ± 29.0 cd	515.1 ± 42.5 d	597.9 ± 34.7 cd	122.8 ± 8.7 d	681.8 ± 62.9 cd	1117.6 ± 95.6 bc	126.2 ± 6.9 b	730.3 ± 58.2 e	87.4 ± 6.7 d	176.1 ± 6.9 c
G2-7	102.3 ± 8.8 d	450.2 ± 20.7 d e	305.4 ± 19.5 d	258.3 ± 13.2 e	155.5 ± 9.9 d	315.6 ± 24.2 b	322.1 ± 9.5 d	555.4 ± 35.4 e	583 ± 24.2 c	72.4 ± 3.5 b	713.4 ± 45.8 d	1067.8 ± 90.8 c	131.1 ± 14.1 b	687.5 ± 81.5 d	84.7 ± 10.3 cd	176.1 ± 9.0 c
G2-10	107.2 ± 6.5 e	459.4 ± 49.2 e	332.2 ± 10.1 e	260 ± 8.4 e	156.6 ± 15.5 d	340.6 ± 21.0 c	340.8 ± 42.1 e	558.8 ± 43.9 e	620.8 ± 73.1 e	32.6 ± 3.1 a	774.1 ± 30.4 e	1213.4 ± 23.5 d	144.6 ± 11.9 c	774.2 ± 81.7 f	80.9 ± 7.6 c	190.6 ± 7.1 d
G3-0	82.4 ± 1.4 a	214 ± 3.2 a	208.8 ± 2.5 c	144.7 ± 3.4 a	97.5 ± 2.1 a	292.3 ± 3.7 c	208.1 ± 2.1 b	330.6 ± 6.2 ab	452.6 ± 8.5 a	104.5 ± 1.9 e	461.8 ± 5.5 d	857.8 ± 17.6 f	103.9 ± 1.2 a	435.7 ± 7.3 d	59.2 ± 1.3 b	124.3 ± 3.4 a
G3-1	86.6 ± 5.8 b	263.3 ± 8.6 b	179.2 ± 12.2 a	186.7 ± 6.2 bc	101 ± 5.8 a	257.1 ± 10.9 a	215.3 ± 7.3 b	314.6 ± 23.0 a	539.6 ± 25.9 bc	107.1 ± 5.8 e	517.9 ± 26.4 e	759.7 ± 52.8 e	129.3 ± 5.2 b	525.6 ± 18.8 e	65.2 ± 2.4 c	150.7 ± 5.5 b
G3-3	119.5 ± 7.5 c	278.1 ± 24.6 b	197.2 ± 18.2 b	190.4 ± 14.5 c	115.6 ± 6.9 b	274.2 ± 14.6 b	232.2 ± 21.0 c	348.3 ± 33.4 ab	550 ± 43.2 c	51.5 ± 2.7 d	370.3 ± 18.6 c	540.9 ± 46.5 d	173.4 ± 9.6 c	439.9 ± 29.3 d	68.7 ± 3.9 d	150.1 ± 6.2 b
G3-5	140.9 ± 10.4 d	315 ± 21.8 c	216.3 ± 13.5 c	175.8 ± 16.9 b	116.2 ± 8.5 b	294.4 ± 14.2 c	183.4 ± 8.6 a	333.4 ± 23.2 b	514.6 ± 34.1 b	42.7 ± 4.1 c	347.5 ± 30.2 b	488.8 ± 21.5 c	235.2 ± 12.5 e	406.5 ± 22.1 c	53.3 ± 4.1 a	119.7 ± 9.5 a
G3-7	170.6 ± 16.4 e	329.8 ± 39.2 c	256.3 ± 11.5 d	203.8 ± 18.8 d	117.1 ± 4.3 b	325.6 ± 16.5 d	211.6 ± 13.9 b	395.3 ± 29.5 c	609.4 ± 39.9 d	23 ± 1.5 a	304.3 ± 34.61 a	445 ± 43.3 b	209.4 ± 13.9 d	367.5 ± 14.7 b	61 ± 3.5 b	142.3 ± 6.5 b
G3-10	171 ± 13.1 e	419.2 ± 15.9 d	250.1 ± 16.4 d	240.9 ± 17.5 e	143.6 ± 11.7 c	357 ± 17.1 e	287.5 ± 16.7 e	424.2 ± 39.6 d	596.5 ± 47.6 e	37.4 ± 1.2 b	293 ± 16.1 a	411.6 ± 48.5 a	223.1 ± 24.5 e	347 ± 16.1 a	85.2 ± 8.2 e	186 ± 7.7 d
G4-0	83.1 ± 1.6 a	218.5 ± 5.1 a	206 ± 2.6 a	146.9 ± 1.8 a	99.1 ± 1.8 a	290 ± 2.8 a	204.7 ± 1.4 a	328.5 ± 2.6 b	449.3 ± 3.0 a	104.1 ± 1.8 f	459.4 ± 6.2 a	857.8 ± 13.1 d	104.9 ± 1.3 a	447 ± 8.3 a	59.2 ± 0.9 a	124.3 ± 3.3 a
G4-1	93.8 ± 5.9 b	282.2 ± 20.2 b	237.3 ± 12.2 b	222.7 ± 8.6 b	124.4 ± 7.5 b	339.5 ± 19.9 b	231.6 ± 10.1 b	288.9 ± 18.8 a	539.4 ± 33.4 c	97.9 ± 6.6 e	615.9 ± 37.3 b	920.8 ± 29.9 e	130.6 ± 8.7 b	567.8 ± 26.7 b	75.5 ± 4.3 c	146 ± 6.2 b
G4-3	137.2 ± 6.8 c	347.9 ± 34.7 c	342.1 ± 34.0 d	242.6 ± 13.9 c	138.3 ± 8.6 c	401.8 ± 32.5 c	253.5 ± 21.9 c	289.8 ± 24.3 a	503.3 ± 23.2 b	64 ± 5.0 d	653.6 ± 44.1 c	517.8 ± 32.8 ab	143.3 ± 9.7 c	603.8 ± 48 cd	87.7 ± 6.7 d	159.8 ± 7.1 bc
G4-5	142.1 ± 14.1 d	389.2 ± 29.2 d	348.4 ± 26.8 d	221 ± 10.7 b	135.4 ± 6.6 c	456 ± 39.5 d	284.8 ± 14.5 d	303.9 ± 26.9 a	497.1 ± 22.0 b	56 ± 4.2 c	661.6 ± 36.1 c d	502.7 ± 31.2 a	131.6 ± 6.5 b	592.2 ± 57 b c	68.3 ± 3.5 b	175.7 ± 7.2 c
G4-7	158 ± 19.1 e	406.2 ± 21.0 d	321.4 ± 35.8 c	240.6 ± 12.2 c	141.9 ± 11.9 c	451.1 ± 30 d	297.8 ± 10.2 e	324.2 ± 21.6 b	521.6 ± 49.7 b c	26.7 ± 1.3 a	689.7 ± 34.0 d	531 ± 17.8 b	143.8 ± 16.9 c	619.2 ± 40.1 d	74.6 ± 4.8 c	213.2 ± 5.5 d
G4-10	169.8 ± 10.0 f	474.5 ± 19.0 e	416.81 ± 19.0 e	275.9 ± 27.6 d	159.6 ± 15.5 d	495.3 ± 53.4 e	317.8 ± 18.2 f	480.2 ± 22.8 c	565.4 ± 54.6 d	37.9 ± 4.6 b	729.6 ± 80.6 e	581.6 ± 28.5 c	157.5 ± 4.6 d	669.2 ± 27.8 e	96.4 ± 5.4 e	199 ± 3.5 d

G1: inoculated with *Corynebacterium glutamicum*; G2: inoculated with *Corynebacterium ammoniagenes*; G3: inoculated with *Lactiplantibacillus plantarum*; G4: inoculated with above three strains. G-FD: Group–Fermentation time(day). In the context of a column, distinct lowercase letters (a–f) were used to denote a statistically significant difference (*p* < 0.05) as determined using Duncan’s test.

## Data Availability

The original contributions presented in the study are included in the article, further inquiries can be directed to the corresponding author.
